# Verrast

**DOI:** 10.1007/s13629-020-00290-y

**Published:** 2020-04-15

**Authors:** Henk G. van der Poel

**Affiliations:** Tijdschrift voor Urologie, Bohn Stafleu van Loghum, Houten, Nederland

Eens temeer worden we verrast. Deze keer door het coronavirus. Wereldwijd nog wel. De situatie vraagt om het snel aanpassen van de urologische patiëntenzorg. Assistenten en urologen die ingezet worden op de IC of worden gevraagd hun zorg op een lager pitje te zetten. Veel vragen bij patiënten, maar ook veel begrip. Iedereen lijkt het belang van een gezamenlijke aanpak in deze viruscrisis in te zien en ook de Nederlandse Vereniging voor Urologie (NVU) werkt aan coördinatie. Duidelijk is wel dat we onvoldoende zijn voorbereid op een pandemie in welke vorm dan ook. Veel hangt af van improvisatie en initiatieven laten zich niet altijd goed integreren. Laten we vooral leren van deze crisis: hoe kunnen we nog beter samenwerken en flexibel blijven. Maar ook leren hoe we de zorg nog efficiënter kunnen maken; dat overleg via internet heel efficiënt en kostenbesparend kan zijn. Kortom waar onze prioriteiten liggen.

Vanwege de coronacrisis gaat ook de voorjaarsvergadering van de NVU niet door. Dat is de reden dat dit nummer van *Tijdschrift voor Urologie* in plaats van met de abstracts gevuld is met vier case reports. Stuk voor stuk over situaties waarin we voor verrassingen gesteld werden en die uiteindelijk vakbekwaam opgelost werden. We komen er doorheen, ook door deze crisis, en we blijven de best mogelijke zorg verlenen aan al onze patiënten. Ook aan hen zonder COVID-19. Ze hebben onze zorg en expertise nodig. Ook nu.

